# Poster 1024: Accelerated healing of full-thickness cutaneous wound using purified apitoxin from asiatic honey bee (*Apis cerana* Fabricius)

**DOI:** 10.1186/1939-4551-7-S1-P14

**Published:** 2014-02-03

**Authors:** Hua Chen, Huili Sun, Jianyu Pan, Bingna Cai, Peng Wan, Deke Chen

**Affiliations:** 1 Chinese Academy of Sciences, South China Sea Institute of Oceanology, CAS Key Laboratory of Tropical Marine Bio-resources and Ecology, Guangzhou, China

## Background

Cutaneous wound healing is a conserved, complex, multi-cellular, multi-molecular process, which is executed and regulated by equally complex signaling networks involving numerous growth factors, cytokines and chemokines. It functions to facilitate barrier restoration following injury-induced loss of skin integrity. Apitoxin, or honey bee venom, has been claimed to be of use in skin wound healing, arthritis, herpes zoster, etc. However, the major allergens in apitoxin such as phospholipase A2 and hyaluronidase, can easily induce life-threatening IgE-mediated allergic reactions in humans. Thus these destructive components should be removed effectively before the apitoxin therapy.

## Methods

In order to test the accelerated wound healing effect of apitoxin from Asiatic honey bee (*Apis cerana* Fabricius), with allergens removed by the 10kDa molecular weight cut-off (MWCO) membrane ultrafitration, full-thickness skin defect of 5×5 mm was constructed on the dorsal area of KM mice. The relative size was measured, and the histological staining of the wound in 3, 5 and 7 days was conducted. The expression of transforming growth factor (TGF)-β1, fibronectin, vascular endothelial growth factor (VEGF) and collagen-I mRNA in the wound healing area was assayed by real time polymerase chain reaction (RT-PCR). And the amount of TGF-β1, fibronectin, VEGF and collagen-I protein was determined by immunohistochemical staining.

## Results

Compared with the control and crude apitoxin groups, the healing rate of full-thickness cutaneous wound was much higher in the purified apitoxin group by 15% at least, with the expression level of TGF-β1, fibronectin and VEGF mRNA decreased, and collagen-I mRNA increased evidently. In addition, the amount of TGF-β1, fibronectin and VEGF protein in the purified apitoxin group was significantly lower than the control and crude apitoxin groups, while collagen-I was much higher by contrast.

## Conclusions

Apitoxin purified by membrane ultrafiltration could enhance the healing process of full-thickness wound in the skin, probably due to its allergic reaction and inflammation symptom decreased effectively. Furthermore, It also played an important role in wound healing, involving complex biological mechanisms associated with the expression of TGF-β1, fibronectin, VEGF and collagen-I, but the underlying mechanism still needs to be further elucidated.

